# A cross-sectional study of insight and family accommodation in pediatric obsessive-compulsive disorder

**DOI:** 10.1186/1753-2000-7-20

**Published:** 2013-06-20

**Authors:** Rajshekhar Bipeta, Srinivasa SRR Yerramilli, Srilakshmi Pingali, Ashok Reddy Karredla, Mohammad Osman Ali

**Affiliations:** 1 Consultant psychiatrist, Rajasri Clinic, Malkajgiri, Hyderabad, Andhra Pradesh, India; 2 Consultant psychiatrist, Sri Venkateswara Nursing Home, Narayanaguda, Hyderabad, Andhra Pradesh, India; 3 Consultant psychiatrist, Roshini Counseling Centre, Somajiguda, Hyderabad, Andhra Pradesh, India; 4 Consultant psychiatrist, Gayatri Clinic, S.R. Nagar, Hyderabad, Andhra Pradesh, India; 5 Consultant psychiatrist, Happiness Medicare, Amberpet, Hyderabad, Andhra Pradesh, India

**Keywords:** Obsessive-compulsive disorder, Child, Adolescent, Pediatric, Insight, Family accommodation

## Abstract

**Background:**

Factors predicting treatment outcome in pediatric patients with obsessive-compulsive disorder (OCD) include disease severity, functional impairment, comorbid disorders, insight, and family accommodation (FA). Treatment of pediatric OCD is often only partly successful as some of these predictors are not targeted with conventional therapy. Among these, insight and FA were identified to be modifiable predictors of special relevance to pediatric OCD. Despite their clinical relevance, insight and FA remain understudied in youth with OCD. This study examined the clinical correlates of insight and FA and determined whether FA mediates the relationship between symptom severity and functional impairment in pediatric OCD.

**Methods:**

This was a cross-sectional, outpatient study. Thirty-five treatment-naive children and adolescentswith DSM-IV diagnosis of OCD (mean age: 13.11 ± 3.16; 54.3% males) were included. Standard questionnaires were administered for assessing the study variables. Insight and comorbidities were assessed based on clinician’s interview. Subjects were categorized as belonging to a high insight or a low insight group, and the differences between these two groups were analyzed using ANOVA. Pearson’s correlation coefficients were calculated for the remaining variables of interest. Mediation analysis was carried out using structural equation modeling.

**Results:**

Relative to those in the high insight group, subjects in the low insight group were younger, had more severe disease and symptoms, and were accommodated to a greater extent by their families. In addition, comorbid depression was more frequent in subjects belonging to the low insight group. Family accommodation was positively related to disease severity, symptom severity, and functional impairment. Family accommodation totally mediated the relationship between symptom severity and functional impairment.

**Conclusions:**

Results support the differences in the diagnostic criteria between adult and pediatric patients with OCD with respect to the requirement of insight. Subjects with low insight displayed clinical characteristics of increased severity compared with their high insight counterparts, suggesting that subjects with low insight may require multimodal approach to treatment. Family accommodation was found to mediate the relationship between symptom severity and functional impairment; the use of family-based approaches to cognitive behavioral therapy, with one of the aims of reducing/mitigating FA, may provide better treatment outcomes in pediatric OCD.

## Background

Obsessive-compulsive disorder (OCD) is a chronic anxiety disorder characterized by the presence of unwanted and recurrent thoughts, ideas, feelings, or mental images (collectively referred to as obsessions) that drive the patient to engage in behaviors or mental acts (referred to as compulsions) designed to prevent or reduce anxiety. OCD occurs not only in adults, but also in children and adolescents and results in substantial distress and functional impairment [1]. Childhood OCD, estimated to affect 1 to 4% of the population [2], is associated with significant multi-domain impairment [3]. This, together with the observation that majority of the adult cases of OCD (up to 80%) have an onset during childhood [4], underscores the importance of early intervention.

Current treatment options for pediatric OCD include cognitive behavioral therapy (CBT), pharmacotherapy, or both. According to the AACAP practice parameters 2012 [5], CBT is recommended as the first-line treatment for mild to moderate cases of OCD in children. In more severe cases, selective serotonin reuptake inhibitors (SSRIs) can be added to CBT. These recommendations are based on the numerous studies that have shown the efficacy and acceptability of CBT, including well-conducted systematic trials [6-10]. A meta-analysis [11] of five randomized controlled trials of CBT in children (N = 161) found a large mean pooled effect size for CBT of 1.45 (95% confidence interval [CI] 0.68–2.22). In addition, CBT has been demonstrated to be effective when delivered individually, or using a family-based or group-setting approach [12-15]. Besides being the first-line treatment for OCD, CBT has other advantages, particularly related to patients with comorbid disorders, for example, comorbid tic disorders were found to adversely impact treatment outcome of SSRIs, but not that of CBT [16]. In addition, group CBT was found to be effective for youth with complex comorbid conditions, including depression, attention deficit/hyperactivity disorder (ADHD) and pervasive developmental disorders (PDD) [12].

Current practice parameters recommend addition of pharmacotherapy to CBT for more severe cases of the disorder. Although addition of pharmacotherapy to CBT confers additional benefit [10,17], many children still fail to respond to the combined treatment and remain symptomatic. In recent clinical intervention studies investigating CBT, pharmacological treatment, or the combination of both in pediatric OCD, results indicated remission rates of 39% with CBT, and from 54% to a maximum of 69% with the combination therapy [10,17]. This emphasizes the need to further investigate the factors that affect treatment outcome and devise novel strategies (based on these factors) for treating pediatric OCD. Among the many factors that were anticipated to be predictors of treatment outcome, OCD severity, OCD-related functional impairment, insight, comorbid externalizing symptoms, and family accommodation (FA) were found to be significant [18]. However, many of these aspects of OCD with the ability to influence treatment response that are particularly relevant in the pediatric OCD context, including comorbid disorders, insight, and family factors, remain understudied. We, therefore, undertook this study to investigate insight and FA as two important modifiable factors associated with pediatric OCD that may serve as critical targets of intervention and to study the interrelations between these factors and, age, duration of illness, sex, comorbidity, disease severity, symptom severity, and functional impairment.

Insight is the recognition of obsessions and compulsions of OCD as unreasonable or excessive. According to the American Psychiatric Association [1], adults can be diagnosed as having OCD only if they have an intact insight into their symptoms. This is in contrast to the requirement in children, who can be diagnosed with OCD even if they have poor insight. Poor insight is recognized as a predictor of worse treatment outcomes in both adult and pediatric OCD [18]. Patients with poor insight, due to their inability to recognize the excessiveness and irrationality of their thoughts, may be less able to challenge their thoughts and less motivated to seek and participate in treatment and, consequently, have worse prognosis [19].

Literature on poor insight is limited in adults and, to a greater extent, in children. Poor insight in adult OCD patients was found to be associated with more compulsions, positive family history of OCD [20], early onset of symptoms, longer duration of illness, increased symptom severity [21] and functional impairment [22], and higher comorbidity, particularly depressive symptoms and schizotypal personality disorder [23,24]. In addition, patients with poor insight had lower metacognition subscale scores [25], impaired neurodevelopment [26] and were found to have difficulty in adequately processing conflicting information, updating their memory with rectified information, and subsequently accessing this corrective information to modify their irrational beliefs [27].

Results of the two main studies that investigated the clinical correlates of insight in pediatric OCD were mildly incongruent to each other. Storch et al. [19] found higher levels of OCD severity, OCD-related functional impairment (parent-rated), and FA in patients with low insight, while no differences were found between the ages of patients with high and low insight. In contrast, Lewin et al. [28] found that insight correlated positively with age. However, insight was found not to be associated with OCD symptom severity, OCD age of onset/illness duration, family history of OCD, parental OCD symptoms, the presence of DSM-IV anxiety/tic/ADHD disorders, and gender. Poorer insight in patients was linked to poorer intellectual functioning and decreased perception of control over their environment, higher levels of depressive symptoms, and lower levels of adaptive functioning. Given that insight in children with OCD needs to be studied further, we planned to investigate the relationship of insight with clinical and family characteristics in pediatric OCD patients. As many questions about insight still remain unanswered (for example, are FA and insight related?), we were also interested in investigating the association between insight and FA.

Family accommodation refers to the actions taken by the family members in facilitating the child’s rituals [29]. Family members may facilitate accommodation of the child’s rituals by avoiding obsessional triggers, getting involved in compulsions, and/or assisting the child in performance of rituals, for example, removing a picture that triggers obsessions, providing reassurance to the child by answering questions repetitively, or helping the child with his/her tasks. In the process of FA, family members unintentionally reinforce the child’s irrational beliefs/ideas. Family accommodation counters the basic rationale of CBT as it circumvents/reduces exposure with response prevention and, thus, prevents the natural habituation of anxiety that develops during the course of therapy and limits the child’s opportunities to learn that the feared consequence is unlikely to occur. In addition, FA also diminishes the aversive consequences of OCD behavior, leading to decreased motivation for change [29].

Only one study has examined the relationship between insight and FA in pediatric OCD patients. Storch et al. [19] reported that parents of youth with low insight endorsed significantly greater levels of FA than parents of youth with higher levels of insight. Family accommodation may lead the child to believe that OCD behavior is reasonable and acceptable. The authors state “Parents of children with poor insight may ‘give in’ and accommodate their children’s behavior after finding that reasoning with them is ineffective.” Since the family plays a central role in the overall development of a child, the role of the family in the development, maintenance, and treatment of pediatric OCD needs to be adequately studied.

In another study, Storch et al. [29] found high rates of FA and significant correlation between FA and, severity of symptoms and child’s functional impairment. They also reported that FA mediated the relationship between symptom severity and functional impairment [29,30]. Peris et al. [31], in contrast, found that FA was not associated with OCD severity, and externalizing and internalizing behavior. Symptom severity was, however, related to parents’ involvement in symptoms. The recognition of FA as an important predictor of treatment response has led to the emergence of family–based treatment for OCD. These approaches need to address critical targets, including reducing FA of symptoms and rituals and augmenting family education, communication, and problem-solving in order to be more effective and associated with long-term maintenance of gains than interventions that target the child alone [32].

The present study was aimed at studying the clinical correlates of insight and FA in pediatric OCD patients and building on the existing data from other studies, in particular, from the studies by Storch et al. [19] and Lewin et al. [28]. In order to understand the focus of intervention among family members and youth with OCD, we also studied the correlations between the study variables and two FA subscales: family accommodation-avoidance of triggers (FAS-AT) and family accommodation-involvement in compulsions (FAS-IC). On the basis of earlier research, we hypothesized that insight is associated with age, duration of illness, symptom severity, OCD severity, functional impairment, and FA. Family accommodation was hypothesized to correlate to disease and symptom severity, functional impairment, and the presence of comorbidities among pediatric OCD patients. Based on the studies conducted by Storch et al. [29] and Caporino et al. [30], we also hypothesized that FA mediates the relationship between OCD symptom severity and functional impairment by reinforcing the child’s irrational behavior by avoiding triggers and getting involved in compulsions, and, consequently, leading to the maintenance of functional impairment related to symptom severity.

## Methods

This was a cross-sectional, clinic-based outpatient study conducted at a psychiatric clinic in Hyderabad, Andhra Pradesh, India. Consecutive and convenience sampling was done. Treatment-seeking subjects and their parents were explained about the nature of the study. Assent was obtained from the subjects, and parents gave written informed consent for participating in the study. After screening, demographic details were collected. Board certified clinical psychiatrists, familiar with OCD diagnostic criteria and standard questionnaires, made the diagnoses using the Schedule for Affective Disorders and Schizophrenia for School-Age Children-Kiddie-SADS-Present and Lifetime Version (KSADS-PL), assessed insight, and disease severity. Children‘s Yale-Brown Obsessive Compulsive Scale (CY-BOCS) was subsequently administered to the child as per the manual. As many pediatric OCD subjects cannot properly estimate their symptoms, both children and parents were interviewed. Specific OCD symptoms were elicited before the 10-item severity ratings. Subsequently, parents completed the Child Obsessive-compulsive Impact Scale-Revised Parent (COIS-RP) and Family Accommodation Scale-Parent Report (FAS-PR), while the subjects completed the Child Obsessive-compulsive Impact Scale-Revised Child (COIS-RC).

### Inclusion criteria

Treatment-seeking and treatment-naive school- or college-going children and adolescents, aged below 18 years, who satisfied the DSM-IV diagnostic criteria of OCD [1], were enrolled. Subjects were included regardless of whether they had completed or interrupted their studies due to illness. Patients and parents who were willing to comply with the study procedures were included.

### Exclusion criteria

Patients with substance abuse/dependence or major medical or surgical illnesses/procedures within the past one year were not included. Those with organic disorders (such as convulsions, complicated head trauma), cognitive impairment, below average intelligence; other Axis I disorders, such as psychotic disorder, bipolar disorder, autistic-spectrum disorder; and current high suicidal tendency were excluded. Parents with below average intelligence, OCD, obsessive-compulsive personality disorder, or any other major psychiatric disorder that would interfere with their ability to comply with study procedures were not interviewed. If one of the parents had OCD, the other parent was interviewed.

### Measures

**Schedule for Affective Disorders and Schizophrenia for School-Age Children-Kiddie-SADS-Present and Lifetime Version (KSADS-PL):** KSADS-PL is a semi-structured interview designed to evaluate DSM-IV psychopathology in the pediatric age group [33].

**Children‘s Yale-Brown Obsessive Compulsive Scale (CY-BOCS):** CY-BOCSis a 10-item semi-structured clinician-rated measure of POCD severity [34]. It has high internal consistency; total score alphas range from 0.87 to 0.90. The CY-BOCS severity scale has been found to have strong convergent and divergent validity [35,36] and is also treatment sensitive [10].

**Child insight:** Insight was assessed using a semi-structured interview by the clinician as described by Lewin et al. [28], who asked the child the following questions, “1) Do you think your problems or behaviors are reasonable (i.e., make sense)? 2) What do you think would happen if you did not perform compulsion(s)? 3) Do you believe that something would really happen?”

Lewin et al. also state, “The clinician was instructed to probe for clarification or additional details. The clinician was instructed to rate the patient’s insight into the senseless or excessiveness of his/her obsessions based on beliefs expressed at the time of the interview using a five point scale: (a) Excellent insight, fully rational; (b) Good insight - readily acknowledges absurdity or excessiveness of thoughts and behaviors but does not seem completely convinced that there is not something besides anxiety to be concerned about (*i.e.,*has lingering doubts); (3) Mild insight – patient may reluctantly admit that thoughts or behaviors seem unreasonable or excessive, but wavers. Patient may have some unrealistic fears, but no fixed convictions; (4) Poor insight – patient maintains that thoughts or behaviors are not unreasonable or excessive, but acknowledges validity of contrary evidence; and (5) Lacks insight, delusional – patient is convinced that concerns and behaviors are reasonable and cannot acknowledge evidence to the contrary” [28].

### Group assignment

Subjects were divided into two groups as described earlier [28]: “low insight group” (children with mild to severe impairment in insight), and “high insight group” (those with excellent or good insight, i.e., without impairment in insight).

**Child Obsessive-compulsive Impact Scale-Revised (COIS-R), Parent and Child report**[37]: The COIS-R is a self-report questionnaire designed to assess pediatric OCD-specific academic, social, and home/family impairment. It has two versions, parent-rated (COIS-RP), and child-rated (COIS-RC).

**Clinical Global Impression-Severity (CGI-S):** CGI-S is a clinician-rated, single-item global Likert-type scale to assess the severity of illness with scores ranging from 1 (“no illness”) to 7 (“serious illness”) [38].

**Family Accommodation Scale-Parent Report (FAS-PR):** The original Family Accommodation Scale (FAS) [39] is a 13-item questionnaire that assesses the degree of FA during the previous month and the level of impairment that the family members and patients experience as a result of FA. Items are scored on a Likert-type 5-point scale. Questions in the FAS assess various areas of accommodation, including the extent to which family members avoid triggers of obsessions and assist in compulsions. For example, questions in the FAS ask parents if they help the child avoid objects, places, or experiences that may cause him/her anxiety, if they provide reassurance to the child or objects needed for compulsions, if they decrease behavioral expectations of the child, or change family activities or routines. Some sample questions from FAS include: (1) “How often did you provide items for the patient’s compulsions?”, (2) “Has the patient become distressed/anxious when you have not provided assistance? To what degree?” The FAS has good psychometric properties [39].

Flessner et al. [40] validated the 12-item version of FAS, called FAS-Parent Report (FAS-PR), and found it to have acceptable convergent and discriminant validity, and internal consistency. According to them, the 12-item version of the FAS is the most appropriate one to use. However, controversy exists regarding which FAS scale is the ideal one to use. Since we wanted to assess the area of focus in family-based treatment approaches, we used the 12-item version of the FAS, FAS-PR, as it provides two subscales: Avoidance of Triggers (FAS-AT) and Involvement in Compulsions (FAS-IC).

### Statistical analysis

Statistical analysis was performed using SPSS v 20.0 (IBM Corp.). Distributions were evaluated for underlying statistical assumptions of the data prior to analyses. Insight was evaluated as a categorical variable. Analysis of variance was used to examine the variables in the low and high insight groups. Associations between the remaining variables were analyzed by Pearson’s correlation. Mediation analysis was carried out using the Baron and Kenny [41] causal steps approach; in addition, a bootstrapped confidence interval for the indirect effect was obtained using AMOS v 20 (IBM Corporation). Overall, 2000 samples were requested, and a bias-corrected confidence interval was created for the indirect path. The initial independent (causal) variable was symptom severity (CY-BOCS) score; the outcome variable was functional impairment-parent reported (COIS-RP) score, and the proposed mediating variable was family accommodation-parent report (FAS-PR).

### Study sample

Of the 42 subjects contacted, parents of four subjects refused to participate. One subject did not meet the inclusion criterion (he was on psychotropic medication). As we wanted to study insight and FA in treatment-seeking and treatment-naive subjects and as insight and FA can change with treatment, we excluded subjects who were on any type of treatment that could affect insight (including psychotropic medication and psychosocial therapies). Two subjects were excluded (one subject was highly suicidal; this subject was excluded because of ethical reasons and for failure to comply with the study requirement of giving written informed consent. Both parents of the second subject had active symptoms of schizophrenia; this subject was excluded because it would have been difficult for the child and parents to comply with the study procedures, including providing written informed consent and filling-up the questionnaires). The final study sample comprised 35 youth [13.11 ± 3.16 years, 54% males (n = 19)]. Table 1 provides the descriptive statistics of the study sample. There were 27 mothers (77%) and 8 fathers (23%). The mean age of parents was 32.51 ± 5.94 years.

**Table 1:** Descriptive statistics of the study variables in pediatric obsessive-compulsive disorder sample (n =35)

**Variable (units)**	**Mean**	**Standard deviation**	**Range**
Age (years)	13.11	3.16	7-17
Age at onset (years)	8.03	1.52	6-11
Duration of illness (months)	60.00	28.51	12-108
CY-BOCS	25.34	9.40	6-38
CY-BOCS-Obsession	11.74	5.86	1-20
CY-BOCS-Compulsion	13.40	5.33	4-20
CGI-S	4.49	1.58	2-7
COIS-RP	54.83	21.80	22-97
CIOS-RC	54.14	22.81	20-96
FAS-PR	27.29	13.64	0-45
FAS-AT	10.09	8.03	0-36
FAS-IC	17.23	8.56	0-34

Note: *CY–BOCS* Children’s Yale–Brown Obsessive–Compulsive Scale, *CGI-S* Clinician’s Global Impression-Severity, *COIS-RP* Child Obsessive-compulsive Impact Scale-Revised Parent report, *COIS-RC* Child Obsessive-compulsive Impact Scale-Child report, *FAS-PR* Family Accommodation Scale-Parent Report, *FAS-AT* Family Accommodation Scale-Avoidance of Triggers, *FAS-IC* Family Accommodation Scale-Involvement in Compulsions.

## Results

### Comorbidity

Of the 35 subjects, at least one comorbidity was present in 14 subjects (40%). Six subjects (17.14%) had multiple comorbid disorders. Depressive disorder was the most common co-occurring disorder in the study population (n = 11; 32%). Other comorbidities included ADHD and conduct disorder.

### Family history of OCD

Three subjects (8.57%) had a family history of OCD. All these three subjects belonged to the high insight group. As the number of subjects with a family history of OCD was extremely small, subgroup analysis was not carried out for this data.

### Insight

**Figure 1:**
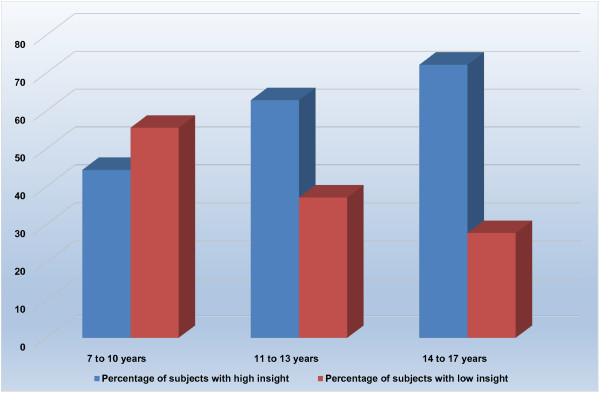
Insight across the age groups.

Of the 35 children, 28 subjects (80%) had high insight, while 7 subjects (20%) had low insight (Chi-square test; p = 0.000). The mean ± SD age in the low insight group was 10.43 ± 3.0 years, and, in the high insight group, was 13.79 ± 2.87 years (t-test; p = 0.010); children with low insight were younger. While only 44.44% of preadolescents (aged 7 to 10 years) had high insight, 62.85% of younger adolescents (aged 11 to 13 years) and 72.22% of older adolescents (aged 14 to 17 years) had high insight. Figure 1 provides the distribution of high and low insight across the three age-groups. Table 2 provides the clinical characteristics of pediatric OCD patients with high and low insight into symptoms.

**Table 2:** Clinical characteristics of pediatric OCD subjects with high and low insight (n =35)

**Variable**	**High insight (n = 28)**	**Low insight (n = 7)**	***t *or Chi-square**	**P value**
Sex (male), n%	16 (48%)	3 (9%)	0.461	0.677
Age	13.79 (2.87)	10.43 (2.99)	7.535	0.010
Duration of illness	63.86 (26.34)	44.57 (33.74)	2.689	0.111
CY-BOCS	27.71 (11.91)	24.75 (8.83)	4.661	0.038
CGI-S	5.29 (1.98)	4.29 (1.44)	11.770	0.002
FAS-PR	31.71 (14.95)	26.18 (13.36)	4.410	0.043
FAS-AT	8.43 (4.47)	10.50 (8.72)	0.365	0.550
FAS-IC	23.29 (11.06)	15.71 (10.07)	3.054	0.090
COIS-RP	63.57 (27.00)	52.65 (20.30)	1.424	0.241
COIS-RC	59.57 (29.86)	52.79 (21.15)	0.488	0.490

Note: *CY–BOCS* Children’s Yale–Brown Obsessive–Compulsive Scale, *CGI-S* Clinician’s Global Impression-Severity, *FAS-PR* Family Accommodation Scale-Parent Report, *FAS-AT* Family Accommodation Scale-Avoidance of Triggers, *FAS-IC* Family Accommodation Scale-Involvement in Compulsions, *COIS-RP* Child Obsessive-compulsive Impact Scale-Revised-Parent report, *COIS-RC* Child Obsessive-compulsive Impact Scale-Revised-Child report.

Depression was found to be significantly more frequent in youth with low insight (57.14%) than in youth with high insight (25%; Chi-square test; p = 0.01). No significant difference was found between the low and the high insight groups for the presence of co-morbid anxiety disorders. Both ADHD and conduct disorder occurred only in the low insight group.

Relative to the subjects with high insight into their symptoms, subjects with low insight were younger (p = 0.010), had more severe OCD (higher CGI-S scores) (p = 0.002), more severe symptoms (higher CY-BOCS scores) (p = 0.038), and were accommodated to a greater extent by family members (higher FAS-PR scores) (p = 0.043). The difference in duration of illness and functional impairment (as measured on COIS), both child- and parent-reported, was not significant between the two groups.

### Family accommodation

### Comorbidity

No statistically significant differences in FA were found in OCD patients with or without comorbidity.

Table 3 presents the correlation matrix for the study variables. Family accommodation (FAS-PR) was significantly related to disease severity (CGI-S), symptom severity (CY-BOCS), parent-reported functional impairment (COIS-RP), and child-reported functional impairment (COIS-RC). Both FAS-AT and FAS-IC were significantly related to disease severity, symptom severity, and parent- and child-reported functional impairment.

**Table 3:** Correlation matrix for the study variables (n = 35)

		**1**	**2**	**3**	**4**	**5**	**7**	**8**	**9**
**1**	**Age (years)**	1							
**2**	**CGI-S**	0.165	1						
**3**	**CY-BOCS**	0.196	0.956**	1					
**4**	**COIS-RP**	0.239	0.954**	0.925**	1				
**5**	**COIS-RC**	0.356*	0.921**	0.910**	0.976**	1			
**7**	**FAS-PR**	0.049	0.897**	0.573**	0.900**	0.879**	1		
**8**	**FAS-AT**	0.387	0.486**	0.573**	0.566**	0.661**	0.631**	1	
**9**	**FAS-IC**	−0.227	0.791**	0.772**	0.734**	0.634**	0.812**	0.060	1

Note: *CY–BOCS* Children’s Yale–Brown Obsessive–Compulsive Scale, *CGI-S* Clinician’s Global Impression-Severity, *COIS-RP* Child Obsessive-compulsive Impact Scale-Revised-Parent report, *COIS-RC* Child Obsessive-compulsive Impact Scale-Revised-Child report, *FAS-PR* Family Accommodation Scale-Parent Report, *FAS-AT* Family Accommodation Scale-Avoidance of Triggers, *FAS-IC* Family Accommodation Scale-Involvement in Compulsions.

Table 4 presents the percentage of parents who endorsed one of the two highest scores on the items in FAS-PR (i.e., 3 or 4 on the item) in pediatric OCD.

**Table 4:** Percentage of parents who endorsed one of the two highest scores on items in FAS-PR* questions (i.e. 3 or 4 on the item)† **in pediatric obsessive-compulsive disorder sample (n = 35)**

FAS-PR* items	**N**	**%**
Providing reassurance	19	54.29
Participating in compulsions	16	45.71
Facilitating avoidance	12	34.29

*FAS-PR = Family Accommodation Scale-Parent Report.

†These items were scored on a scale of 0 (never), 1 (once/week), 2 (2–3 times/week), 3 (4–6 times/week), 4 (every day).

### Mediation analysis

**Figure 2:**
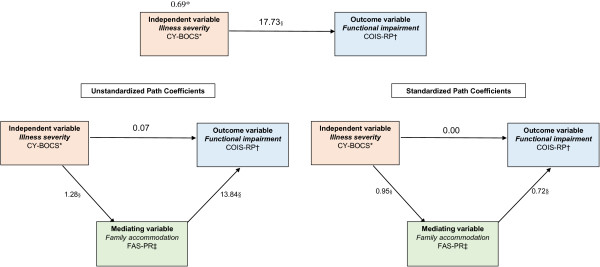
**Path diagram for the mediation model (n = 35).** Standardized and unstandardized path coefficients are reported. *CY-BOCS = Children’s Yale-Brown Obsessive-Compulsive Scale; †COIS-RP = Child Obsessive-compulsive Impact Scale, Revised-Parent report;‡FAS-PR = Family Accommodation Scale-Parent Report *Standardized coefficient for the total effect. §Correlation is significant at the 0.001 level (2-tailed).

As described previously [42], mediation is demonstrated when (i) the independent variable significantly correlates with the dependent variable, (ii) the independent variable is significantly related to the mediator variable, (iii) the mediator variable has a unique effect on the dependent variable when the independent variable is controlled, and (iv) the effect of the independent variable on the dependent variable diminishes significantly when the mediator is added. In this study, the independent variable (symptom severity; CY-BOCS) significantly correlated with, the dependent variable (parent-reported functional impairment; COIS-RP) and the mediator variable (family accommodation scale-parent report; FAS-PR). Preliminary data analysis suggested no serious violations of assumptions of normality or linearity. In our model, because each variable had a direct path to every other variable, the chi- square for model fit was 0 (this means that the path coefficients could perfectly reconstruct the variances and covariances among the observed variables). All coefficients reported here are b = unstandardized, Beta = standardized, unless otherwise stated; if the bootstrapped confidence did not include zero for the indirect effect, significance was considered to have been achieved. Figure 2 depicts the path diagram corresponding to this mediation hypothesis.

The total effect of CY-BOCS on COIS-RP was significant, b = 17.73 (CI: 12.23–24.15; p = 0.001), Beta = 0.69 (CI: 0.48–0.80; p = 0.002); each 1-score increase in CY-BOCS predicted approximately 17.7-point increase in COIS-RP score. CY-BOCS was significantly predictive of the hypothesized mediating variable, FAS-PR;b = 1.28 (CI: 1.10–1.43; p = 0.001), Beta = 0.95 (CI: 0.92–0.97; p = 0.003). When controlling for CY-BOCS, FAS-PR was significantly predictive of COIS-RP, b = 13.85 (CI: 2.14–31.36; p = 0.009), Beta = 0.72 (CI: 0.12–1.79; p = 0.007). The estimated direct effect of CY-BOCS on COIS-RP, controlling for FAS-PR, was b = 0.073 (CI: 29.23–17.25; p = 0.982), Beta = 0.003 (CI: -1.15–0.64; p = 0.980).

COIS-RP was predicted well from CY-BOCS and FAS-PR, with adjusted *R*^*2*^ *=* 0.69 (CI: 0.46–0.99; p = 0.000).

The indirect effect was b = 17.66 (CI: 2.90–43.00; p = 0.008), Beta = 0.68 (CI: 0.12–1.76; p = 0.007). This was judged to be statistically significant using the 95% CI, as it did not contain zero. Thus, the indirect effect of CY-BOCS on COIS-RP through FAS-PR was statistically significant. However, the direct path from CY-BOCS to COIS-RP was not statistically significant; therefore, the effects of CY-BOCS on COIS-RP were totally mediated by FAS-PR.

The left-hand side diagram in Figure 2 shows the unstandardized path coefficients for this mediation analysis; the right-hand side diagram shows the corresponding standardized path coefficients.

Comparison of the coefficients for the direct versus the indirect paths0.07 vs. 17.66 suggests that a relatively large part of the effect of CY-BOCS on COIS-RP is mediated by FAS-PR. However, there may be other mediating variables through which CY-BOCS might influence COIS-RP.

## Discussion

The primary aim of this study was to understand the clinical correlates of insight and FA in a representative sample of the pediatric OCD population to be treated, as limited data exist on clinical characteristics as a function of insight, although insight has been recognized as an important clinical characteristic of OCD. We enrolled treatment-naive subjects in the study; this criterion may limit the generalizability of our findings given that such a sample may not be representative of youth who present to the clinic for OCD treatment. However, considering the fact that this study was conducted in a developing country (i.e. India) and recruitment of the subjects was only subtly affected by this criterion, it seems likely that a fraction of the OCD patients, particularly in countries without centrally managed health care, remain treatment-naive at presentation and this may vary from country to country.

Consistent with literature published earlier [28]; we found significant associations between insight and age, and insight and co-morbid depression. In agreement with earlier findings [19], low insight in subjects was found to be significantly associated with disease severity, symptom severity, and FA. Our findings are different from those of Lewin et al. [28] in that they did not find group differences in OCD-symptom severity, and from those of Storch et al. [19], as we did not find significant differences between both parent-reported and child-reported functional impairment in the two insight groups. However, since the sample size of our study was small, and the number of subjects in the low insight group even smaller, the inconsistency in finding can be an artifact of the small sample. Overall, these findings suggest that the clinical presentation of children with low insight is distinct than from those with high insight, with increased disease severity, symptom severity, and the presence of comorbid conditions, particularly depression in patients with low insight.

A significant finding in our study was the relationship between insight and age of the subjects. However, the relationship between duration of illness and insight was not significant. It may be that, because of the cognitive and neurodevelopmental differences, subjects who are younger tend to have low insight into their symptoms. As described by Piaget [42], the development of insight takes place along with the emergence of abstract thinking/formal operations during the period of transition into adulthood. Younger children may therefore have low insight into their symptoms.

Our study supports the diagnostic differences in insight with respect to age between adults and youth with OCD. If the diagnostic criteria of adults are applied to children, OCD diagnoses may be missed in a number of patients with poor insight; this may have clinical and prognostic implications considering that younger children have poorer insight and early intervention may help in preventing impairment and negative effects on development.

Parents of youth with low insight endorsed higher levels of FA than did parents of patients with high insight. There may be two reasons why parental accommodation is high in patients with low insight. One, parents may find reasoning with children with low insight to be futile or ineffective and, therefore, may give in to ritualistic demands. On the other hand, children with OCD may start to “view” their OCD behaviors as normal due to parental accommodation and lack of resistance. In either case, parental accommodation “reinforces” the impairment in insight. Since lack of insight may result in less resistance to obsessive-compulsive symptoms, which is vital for successful CBT, children with low or impaired insight may be more resistant to treatment and have worse prognosis.

In keeping with earlier studies [29,31], parents reported high rates of FA (54.29% of parents endorsed one of the two highest scores on items in the FAS pertaining to providing reassurance, 45.71% of parents endorsed one of the two highest scores on items in the FAS pertaining to participating in compulsions, and 34.29% of parents endorsed one of the two highest scores on items in the FAS pertaining to facilitating avoidance), most frequently reassuring their children, participating in rituals, and assisting in avoidance. In this pediatric study, FA and both subscales of FAS, i.e., FAS-AT and FAS-IC, were positively related to child functional impairment. FA and the two subscales were significantly related to both symptom severity and parent-reported functional impairment. As hypothesized, FA mediated the relation between OCD symptom severity and parent-rated child functional impairment. Our study builds on the earlier two studies by Storch et al. and Caporino et al. [29,30] who also found that FA mediates the relationship between symptom severity and functional impairment and, thus, our study underscores the role of the family in treatment of pediatric OCD. Since family members are also responsible in maintaining OCD symptoms and functional impairment, these interactions need to be addressed to ensure optimal treatment gains. One important difference between the findings from the Storch study [29] and our study relates to the strong correlation between symptom severity and child-reported functional impairment, and parent-reported family accommodation with child-rated functional impairment. Both these were significantly related in our study, but not in the one by Storch. They speculated that parents may more consistently associate impairment with greater symptom severity, whereas children may be more variable in their reports and, alternatively, children with severe symptoms may experience less subjective distress and impairment due to significant FA [29]. Our study suggests that children were as consistent as parents in reporting on symptom severity and functional impairment, and that both parents and children viewed functional impairment and FA as corresponding to severity of symptoms. However, since the FAS-PR is not a validated scale in India, this finding needs to be appreciated with caution.

Our study is unique in that (i) to the best of our knowledge, no published original research study used a 12-item version of FAS or its subscales, FAS-P-AT (Avoidance of Triggers) and FAS-P-IC (Involvement in Compulsions), which have a role in the etiology, maintenance, and treatment of pediatric OCD, (ii) we also examined for differences in FA based on comorbidity, which was not done in previous published studies.

Apart from the small sample size, this study has certain other limitations: (i) The investigators were not blinded to the study procedure. Younger children may have been rated as having lower insight due to interviewer bias; (ii) Children may have developmental differences, for example, problems with expressing themselves because language skills would still be developing, and children were not matched for age in the low and high insight groups; (iii) Many of the assessment instruments have not been standardized for the Indian population. The measures were neither validated nor translated in relevant languages. We did not establish inter-rater reliability on measures, including that relating to insight; and (v) This was a clinic-based cross-sectional study treatment-naive on school/college-going treatment-naive subjects. Therefore, the results may not be generalizable to pediatric OCD patients in the community.

Through our study, we have tried to gain insight into the clinical characteristics of pediatric OCD patients. However, much scope for research exists in this subset of the OCD population on hitherto unexplored aspects, including (i) The assessment of the relationship of insight with specific obsessions and compulsions; (ii) The assessment of the development of insight as the child grows, (iii) Insight assessment instruments specific to pediatric age group need to be developed; and (iv) Theinfluence of bio-psycho-social interventions on insight need to be studied.

There is a need to validate the FAS-PR and other scales for the Asian population, especially the Indian population. Increasing importance needs to be given to involving the family in the treatment of pediatric patients with OCD in these populations. However, the content of CBT remains to be tailored to the requirements of the population to be treated, and the effectiveness of the devised content to be investigated.

## Conclusion

This study provides support to the difference in the criterion for insight in DSM diagnosis of OCD among adult and pediatric patients of OCD. Younger children may have poor insight, and the requirement of an intact insight may cause OCD diagnosis to be missed in younger pediatric patients. As suggested earlier, pediatric OCD with low insight may represent a distinct clinical subtype in that it is associated with increased disease and symptom severity. Family accommodation is also greater in patients with low insight. Family accommodation is positively related to disease severity, symptom severity, and functional impairment, indicating that families of pediatric patients with more severe disease and symptoms accommodate the disorder to a greater degree. As FA is a mediator of functional impairment and a significant predictor of treatment outcome, involving the family in the child’s OCD treatment may provide better outcomes to treatment.

## Abbreviations

CBT: Cognitive behavioral therapy; CGI-S: Clinical Global Impression-Severity; COIS-R: Child Obsessive-compulsive Impact Scale-Revised, Parent (COIS-RP) and COIS-RC, Child report; DSM-IV: Diagnostic and Statistical Manual of Mental Disorders, 4th edition; FA: Family accommodation; KSADS-PL: Schedule for Affective Disorders and Schizophrenia for School-Age Children-Kiddie-SADS-Present and Lifetime Version; OCD: Obsessive-compulsive disorder

## Competing interests

The authors declare that they have no competing interests.

## Authors’ contributions

RB was involved in conceptualizing and designing the study, and was a major contributor in preparing and writing the manuscript. RB and SSRRY acquired the data and performed all the assessments. RB, SSRRY, SP, KAR, and MOA analyzed and interpreted the data. All the authors were involved in revising and editing the manuscript critically for important intellectual content. They have read and given approval for the final version of the manuscript to be published. All the authors made substantive intellectual contributions to this study, and participated sufficiently in the work, and take public responsibility for appropriate portions of the content. All authors read and approved the final manuscript.

## Authors’ information

RB has hands on experience with various assessment schedules and rating scales, and this is one of his major areas of interest. He routinely uses these in his clinical practice.
